# Spatial and temporal analysis on the impact of ultra-low volume indoor insecticide spraying on *Aedes aegypti* household density

**DOI:** 10.1186/s13071-024-06308-3

**Published:** 2024-06-11

**Authors:** Anna B. Kawiecki, Amy C. Morrison, Christopher M. Barker

**Affiliations:** 1 University of California Davis, Davis, CA USA; 2 Pacific Southwest Center of Excellence in Vector-Borne Diseases, University of California Davis, Davis, CA USA

**Keywords:** *Aedes aegypti*, Dengue, Mosquito, Vector-borne diseases, Insecticide, Insect control, Peru

## Abstract

**Background:**

*Aedes aegypti* is the primary mosquito vector for several arboviruses, such as dengue, chikungunya and Zika viruses, which cause frequent outbreaks of human disease in tropical and subtropical regions. Control of these outbreaks relies on vector control, commonly in the form of insecticide sprays that target adult female mosquitoes. However, the spatial coverage and frequency of sprays needed to optimize effectiveness are unclear. In this study, we characterize the effect of ultra-low-volume (ULV) indoor spraying of pyrethroid insecticides on *Ae. aegypti* abundance within households. We also evaluate the effects of spray events during recent time periods or in neighboring households. Improved understanding of the duration and distance of the impact of a spray intervention on *Ae. aegypti* populations can inform vector control interventions, in addition to modeling efforts that contrast vector control strategies.

**Methods:**

This project analyzes data from two large-scale experiments that involved six cycles of indoor pyrethroid spray applications in 2 years in the Amazonian city of Iquitos, Peru. We developed spatial multi-level models to disentangle the reduction in *Ae. aegypti* abundance that resulted from (i) recent ULV treatment within households and (ii) ULV treatment of adjacent or nearby households. We compared fits of models across a range of candidate weighting schemes for the spray effect, based on different temporal and spatial decay functions to understand lagged ULV effects.

**Results:**

Our results suggested that the reduction of *Ae. aegypti* in a household was mainly due to spray events occurring within the same household, with no additional effect of sprays that occurred in neighboring households. Effectiveness of a spray intervention should be measured based on time since the most recent spray event, as we found no cumulative effect of sequential sprays. Based on our model, we estimated the spray effect is reduced by 50% approximately 28 days after the spray event.

**Conclusions:**

The reduction of *Ae. aegypti* in a household was mainly determined by the number of days since the last spray intervention in that same household, highlighting the importance of spray coverage in high-risk areas with a spray frequency determined by local viral transmission dynamics.

**Graphical abstract:**

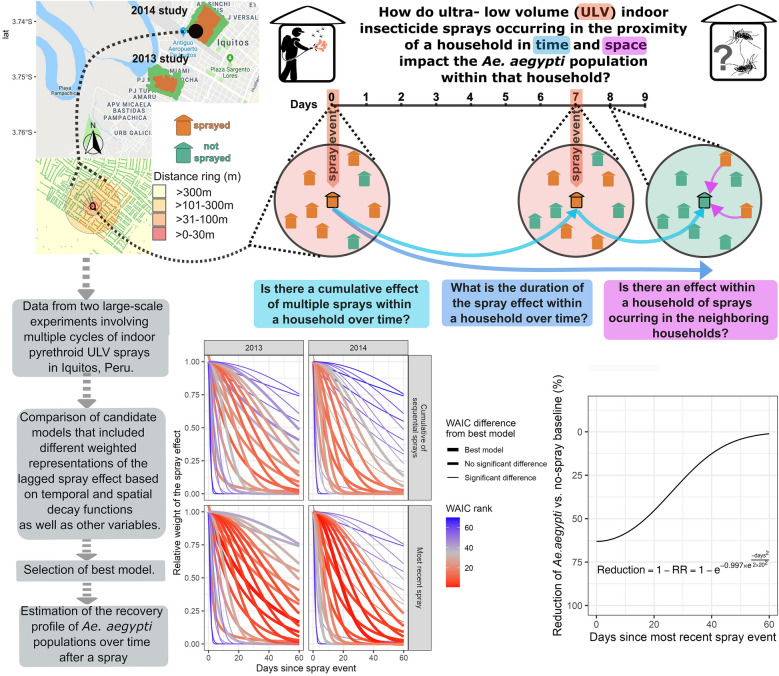

**Supplementary Information:**

The online version contains supplementary material available at 10.1186/s13071-024-06308-3.

## Background

*Aedes aegypti* is the main vector for several arboviruses that can cause widespread epidemics, including dengue (DENV), chikungunya and Zika viruses. This mosquito species feeds primarily and frequently on humans and is well-adapted to urban environments [1–4], where it has become established in many regions throughout the tropics and subtropics [5]. In many of these areas, dengue outbreaks recur periodically, resulting in around 390 million cases per year [6, 7]. Due to the lack of treatment or effective, widely distributed vaccines, prevention and control of DENV transmission rely on reducing mosquito populations through various vector control measures, commonly as insecticide sprays that target adult mosquitoes [8].

In many tropical areas, indoor sprays are more effective at reducing *Ae. aegypti* populations than outdoor sprays, as these mosquitoes commonly rest and bite indoors where indoor habitats are readily accessible [9–12]. Ultra-low-volume (ULV) indoor insecticide spraying consists of applying aerosols with the minimum effective volume of undiluted insecticide product inside structures [12, 13] and is widely used to control outbreaks in the tropics [12, 14]. Indoor ULV spray interventions can be effective, but are expensive and require trained personnel and calibrated instruments [15–17]. In addition, guidelines regarding the spatial and temporal coverage of households required for the intervention to be effective are unclear and lack quantitative evidence, as there are few studies that describe the effectiveness of spray events over time and space in a field setting [8, 14, 18–21]. Field studies that evaluate the effectiveness of vector control measures pose a logistical and economic challenge because of site-specific dynamics of mosquito populations, viral strains, characteristics of the built environment and environmental conditions [13]. Models can assist in evaluating and comparing the effectiveness of vector control strategies on vector and virus transmission reduction using location-specific parameters but need to be informed with real-world data.

In this study, we used data from two large field experiments of repeated ULV indoor pyrethroid spray applications in the city of Iquitos in the Peruvian Amazon [14] to evaluate the spatial and temporal lagged effects of ULV spray events occurring beyond the individual household on the abundance of *Ae. aegypti* per household. A previous study estimated effects of ULV treatments based on whether households were inside or outside of broad blocks that received the intervention. In this study, we aimed to disaggregate the treatment effects at the finer scale of individual households to understand the relative contributions of treatment within a household compared to treatments in neighboring households. Temporally, we evaluated the cumulative effect of repeated sprays vs. the effect of the most recent spray on *Ae. aegypti* reduction in a house to understand the required frequency of sprays and to provide an estimate for the decay of the spray effect over time. This analysis can inform vector control strategies and provide information to parameterize models to predict their effectiveness [22–24].

## Methods

### Study description

Data for this study were collected during two field experiments involving six cycles of indoor ULV pyrethroid spray interventions in the city of Iquitos, Peru. The study area, intervention and data collection methods have been described previously [14]. Briefly, the two experiments were similar but took place in different years (2013 and 2014) and different areas of the city and had slightly different study designs and implementation. Henceforth, we denote the two studies by year as S-2013 and L-2014. Both study areas included a central zone that received the spray applications, or “spray zone,” and a surrounding “buffer zone” that was unsprayed. Within spray zones, treatment status was tracked at the level of individual households. Adult *Ae. aegypti* collections (henceforth adult surveys) were performed using Prokopack aspirators across the entire study area before, during and after the spray interventions [14] using a standardized sampling protocol per household as previously described [14, 25, 26]. Pyrethroid insecticides were applied in six cycles over 6 weeks using Stihl or Solo backpack sprayers that had been adjusted for ULV application or hand-held Colt ULV sprayers [14]. In S-2013 the pyrethroid applied was alphacypermethrin 10% (Turbine 10%), and adult surveys were performed before each spray event; thus, most adult surveys took place 5–8 days after a spray intervention [14]. In L-2014, the insecticide applied was cypermethrin 20%, and adult surveys generally took place 1–4 days after a household was sprayed. Furthermore, in L-2014 an emergency spray intervention of a different formulation of cypermethrin 20% was applied by the Ministry of Health (MoH) using Solo backpack sprayers in both the spray and buffer zones. Data were projected in Universal Transverse Mercator, Zone 18S, WGS1984 datum, that corresponds to the zone where Iquitos, Peru, is located.

### Statistical analysis

The outcome of interest was defined as the total number of *Ae. aegypti* adults collected, ${y}_{it}$, per household *i* and time *t*, which was modeled in a multi-level Bayesian framework using a negative binomial distribution to account for overdispersion, especially because of the large number of collections with zero adults [14, 27]. All candidate models were fitted separately to data sets for S-2013 and L-2014, given the differences in location and experimental design between the two studies. Candidate models were developed following the general form:

1 $$\begin{array}{c}{y}_{it}\sim NB\left({\mu }_{it},\theta \right)\end{array}$$

2 $$\begin{array}{c}log\left({\mu }_{it}\right)={\alpha }_{0}+{\beta }_{a}{a}_{it}+{\beta }_{b}{b}_{it}+{\gamma }_{i}+{\delta }_{t}+{\eta }_{i}\end{array}$$

where

- ${\mu }_{it}$ represents the mean of the negative binomial distribution
- $\theta$ represents the overdispersion parameter
- *i* represents the location (i.e. house) of the adult survey:
  - for S-2013 *i* = 1,2,…,1220
  - for L-2014 *i *= 1,2,…,2182
- *t* represents the time point (i.e. date) of the adult survey
  - for S-2013 *t* = 1,2,…,48 between 2013–04-22 and 2013–08-08
  - for L-2014 *t* = 1,2,…,132 between 2014–01-07 and 2014–11-07
- ${\alpha }_{0}$ represents the overall intercept corresponding to the average number of adult *Ae. aegypti* per unsprayed household across all locations and time points
- ${\gamma }_{i}$ represents a random effect that accounts for spatial autocorrelation following a zero-mean Gaussian process with a Matérn covariance function computed using SPDE (stochastic partial differential equation)[28–30].
- ${\delta }_{t}$ represents a random effect according to a random walk of order one (RW1) for the month of *t* to account for seasonal trends [31]
- ${\eta }_{i}$ represents an independent, identically distributed (iid) random effect that accounts for baseline abundance of each household *i*
- *a* represents any of a set of candidate variables that measure the effect of spraying household *i* at time *t*, described below (Additional file 1: Table 2 and Table 3).
- *b* represents any of a set of candidate variables that measure the effect of spraying the neighboring households surrounding household *i* at time *t*, described below (Additional file 1: Table 4 and Table 5).

Models were fitted using the R-INLA package in R that employs the integrated nested Laplace approximation (INLA) method for Bayesian inference [32]. Prior distributions for parameters were chosen with the goals of being weakly informative and parsimonious, using the defaults proposed in the R-INLA documentation. We assumed zero-mean Gaussian prior distributions $\beta \sim Normal\left(0,{0.001}^{-1}\right)$ for all fixed effects (*a* and *b*) and log gamma prior distributions for the iid and RW1 random effects (${\eta }_{i}\sim loggamma\left(1,0.00005\right)$ and ${\delta }_{t}\sim loggamma\left(1,0.00005\right)$). For the spatial random effect, we implemented a penalized complexity (PC) prior with $range=c\left(10,0.01\right)$, and $\sigma =c\left(1,0.01\right)$. PC priors are recommended, as they reward simplicity by shrinking parameter estimates toward a “base model,” thus preventing overfitting [33]. To ensure that the results (WAIC and rate ratios) were robust to prior choices, we performed a prior sensitivity analysis with 13 alternative priors for the fixed and random effects (Additional file 1: Table 1, Additional file 1: Fig. S1), further described in section 1 of Additional file 1.

#### Within-household spray effects

To measure the effect of a spray event in household *i* (*a*) we compared 68 candidate models that included different weighted representations of the spraying histories within the household, where we were mainly interested in measuring the temporal duration of the spray effect. Candidate models followed the form of Eq. 2 above where variable *a* was one of the 68 candidate variables (Additional file 1: Table 2 and Table 3) and variable *b* was 0. Variables varied in complexity from binary indicators of treatment status (Additional file 1: Table 2) to more nuanced measures that assigned a weighted value to the time since a spray event or events based on an array of candidate decay functions (Additional file 1: Table 3). Discrete and continuous measures of *a* are fully described in Additional file 1: Table 2 and include an array of yes/no variables that indicated whether household *i* had been sprayed in the previous 1–6 weeks, as both a single variable (example: *a* = *sprayed 1 week prior*) and as a combination of variables (example: *a* = *sprayed 1 week prior* + *sprayed 2 weeks prior*).

**Fig. 1:**
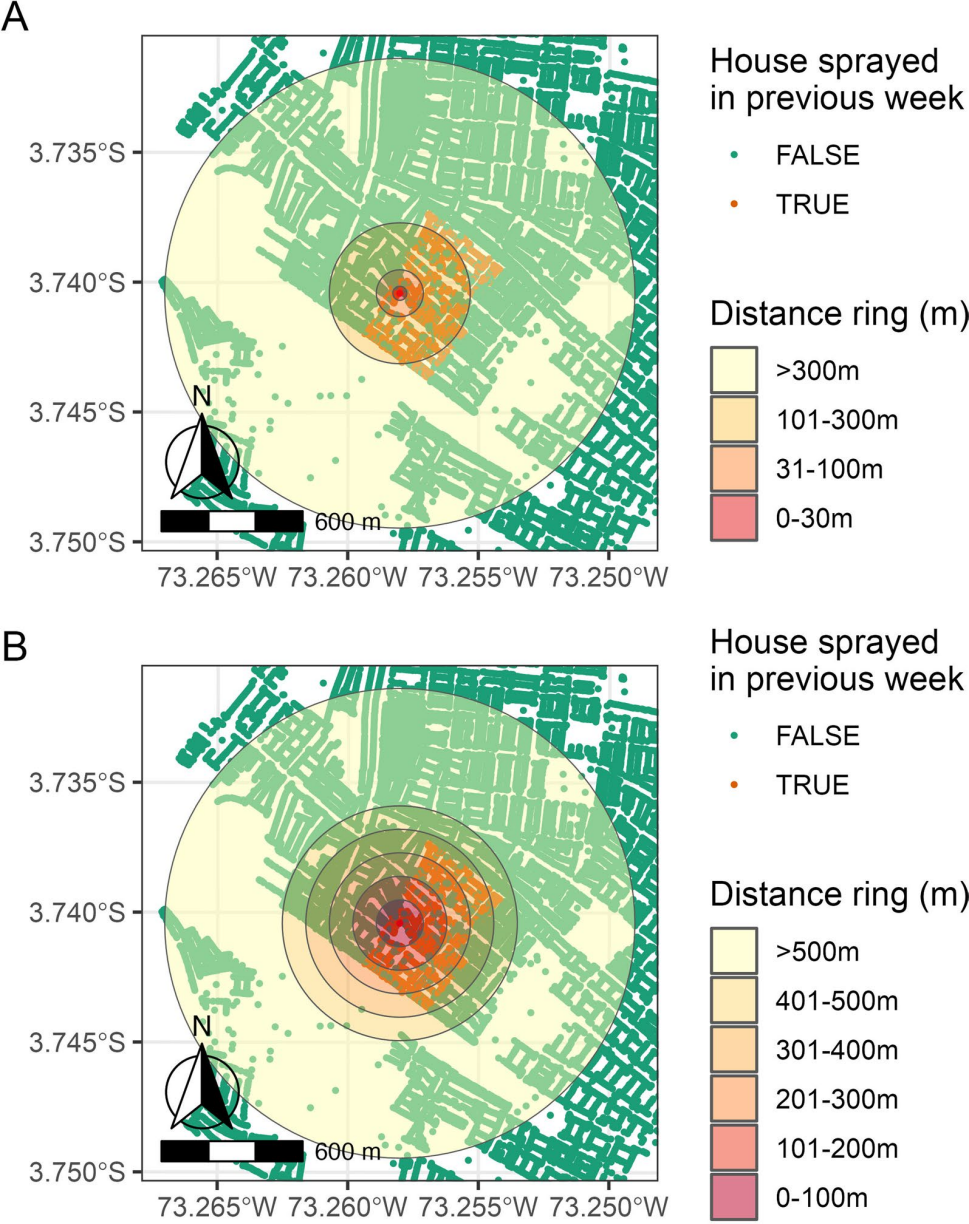
Visual representation of the ring distance schemes used to calculate the proportion of households within a ring of a given distance from household *i* that had been sprayed in the week before *t* (all within a buffer of 1000 m from each household *i*). In this example from L-2014, household* i* is located in the spray zone, and the adult survey occurred after the second cycle of sprays. **A** The distance rings are based on the distance *Aedes aegypti* have been reported to fly. **B** The distance rings are based on an even spacing every 100 m

Variables measuring continuous time since spray events were calculated based on the difference in days between the date of the adult survey *t* and the date of a spray event that occurred prior to *t*, denoted as *s* (Eq. 3). A value of one was added to the difference in days between *t* and *s* so that the result would have a positive value, including when the spray events occurred on the same day as the adult survey; the resulting value is denoted as$\Delta t$. In S-2013 adult mosquito surveys occurred prior to the spray event; thus, adult surveys occurring on the same day as a spray event were not accounted for, as that spray event could not have had an effect on the number of adult mosquitoes in the household [14]. Because there were six cycles of spray interventions, for every household *i* and time point *t*, there were up to six values of$\Delta t$, denoted as $\Delta {t}_{c}$, where *c* represents the cycle number ($c=1,2,\dots 6$).

3 $$\begin{array}{c}\Delta {t}_{c}=t-{s}_{c}+1\end{array}$$

For a more detailed representation of the duration of the effect of a spray event in household *i*, we created candidate variables that assigned a weight to $\Delta {t}_{c}$ based on a decay function, $f\left(\Delta {t}_{c}\right)$ (Additional file 1: Table 3). The different functions tested, representing different possible decay rates of the spray effect, were the inverse, Gaussian and exponential functions given by (Additional file 1: Fig. S2):

Inverse function:

4 $$\begin{array}{c}f\left(\Delta {t}_{c}\right)=\frac{1}{\Delta {t}_{c}}\end{array}$$

Gaussian function:

5 $$\begin{array}{c}f\left(\Delta {t}_{c}\right)={e}^{-\frac{\Delta {t}_{c}^{2}}{2\times {\sigma }^{2}}}\end{array}$$

where $$\sigma =1,3,5,7,10,15,20,25,30,35,40,50,60,80$$

Exponential function:

6 $$\begin{array}{c}f\left(\Delta {t}_{c}\right)={e}^{\left(k\times \Delta {t}_{c}\right)}\end{array}$$

where $$k=0.005,0.010,0.015,0.02,0.03,0.04,0.06,0.1,0.2,0.4,1$$

For each function we designed two variables:

1. the weighted value of the most recent spray
  7 $$\begin{array}{c}{a}_{{\text{it}}}=max\left(f\left(\Delta {t}_{c}\right)\right)\end{array}$$
2. the cumulative value of the weights of all previous spray events
  8 $$\begin{array}{c}{a}_{it}=\sum_{c=1}^{6}\left(f\left(\Delta {t}_{c}\right)\right)\end{array}$$

Candidate models were compared using the widely applicable information criterion or Watanabe-Akaike information criterion (WAIC) whereby the models with lowest WAIC values were considered to have the best fit [34, 35]. In addition, we compared models by calculating the difference in WAIC between each candidate model and the model with lowest WAIC (dWAIC) as well as an approximate measure of the uncertainty around the dWAIC. Models with an uncertainty interval that overlapped zero were considered to not have significantly different fit from the best fitting model. Comparisons were performed separately between the models fit to either the 2013 or 2014 data set. To select the model that best represented *a* overall, we chose the model that performed the best when applied to both data sets, that is, the model with the lowest WAIC common to the two. To do this we averaged the model WAIC rank between the two experiments and chose the model with the best average WAIC rank. The model selected using this process will be denoted ${m}_{best}$.

#### Effects of sprays in neighboring households

To estimate any additional effect of spraying the households surrounding household *i* on the number of adult *Ae. aegypti* in household *i* (*b*), we compared various candidate models that followed the form of Eq. 2 above. In these models, variable *a* was the variable *a* selected in ${m}_{best}$ described above, and variable *b* was one of the 30 candidate *b* variables (Additional file 1: Table 4 and Table 5). The variables measuring *b* were calculated based on the distance (in m) between the household *i* and every surrounding household *j*, or ${d}_{ij}$.

We tested simple measures of *b* by calculating the proportion of households within a ring of a given distance from household *i* that had been sprayed in the week before *t* (Additional file 1: Table 4).

9 $$\begin{array}{c}{b}_{i{t}_{r}}=\frac{{h}_{sprayed,r} \times 10}{{h}_{total,r}}\end{array}$$

where *h* is the number of households in ring *r*, and *r* is the distance ring from household *i*. Ring distances were assigned based on:

1. biological plausibility, given the distance that *Aedes* mosquitoes have been recorded to fly in the literature [36–40] (Fig. 1A): 1–30 m, 31–100 m, 101–300 m, > 300 m.
2. an even distribution of distance spacing (Fig. 1B): 1–100 m, 101–200 m, 201–300 m, 301–400 m, 401–500 m, > 500 m.

Calculations for the proportion included in the denominator houses up to 1000 m from each household *i*; thus, the distance ring > 300 m is 300 m–1000 m and > 500 m is 500 m–1000 m.

In addition, we tested variables that represented different possible decay rates of the spray over space by assigning weights to ${d}_{ij}$ using a series of decay functions, $f\left({d}_{ij}\right)$ (Additional file 1: Table 5). As described above, the decay functions used were the inverse, Gaussian and exponential functions; however, for effect *b* the varying decay rate parameters were: for the Gaussian function $\sigma =5, 25, 50, 75, 100, 125, 150, 200, 250, 300$; for the exponential function $k=0.0025, 0.0035, 0.005, 0.0075, 0.01, 0.0125, 0.02, 0.045, 0.2$ (Additional file 1: Fig. S3). The variables measuring *b* also accounted for spray events in household *j* prior to *t* by multiplying the weighted measure of distance, $f\left({d}_{ij}\right)$, by the weighted measure of “time since spray event” using the weight selected in the previous step, ${f}_{{m}_{best}}\left(\Delta {t}_{c}\right)$, for every household *j*. The resulting weight was added for all households surrounding household *i*.

10 $$\begin{array}{c}{b}_{it}=\sum_{j}\left(f\left({d}_{ij}\right)\times {f}_{{m}_{best}}\left(\Delta {t}_{c}\right)\right)\end{array}$$

Models were compared and selected similarly to what was described above for fixed effect *a*, with the difference that the WAIC of each candidate model was compared to the WAIC of ${m}_{best}$.

## Results

### Within-household spray effects

**Fig. 2:**
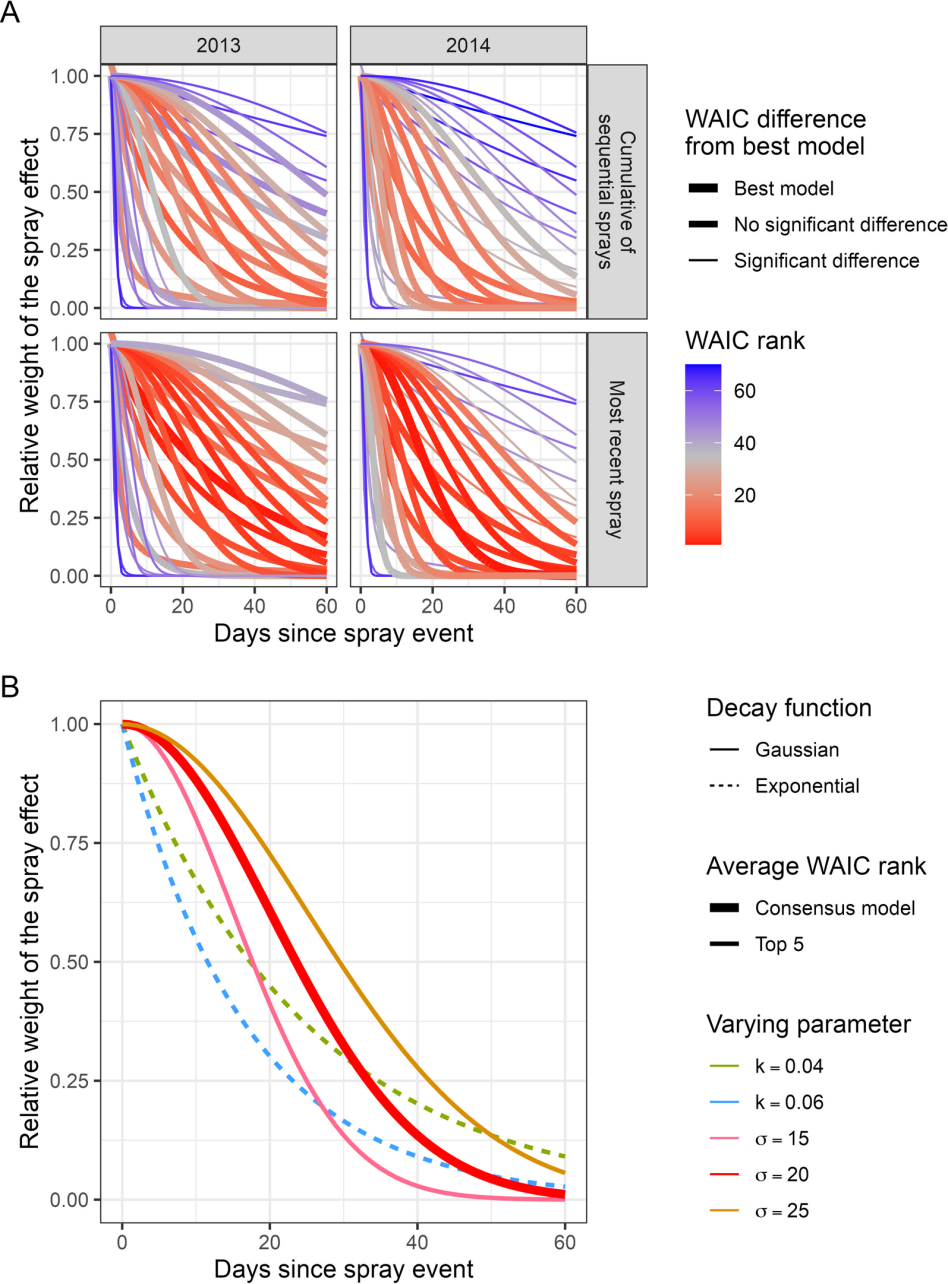
**A** Relative model fits of within-household temporal weighting functions for spray effects. Thicker red lines indicate best-fitting models, with the thickest line indicating the best-fitting model and the other thick lines representing models where the WAIC was not significantly different from the WAIC of the best fitting model. **B** Decay functions applied to the days since most recent spray that were within the top five best-fitting models by average WAIC rank across the two experiments

To evaluate the effect of a spray event in household *i* on the number of adult *Ae. aegypti* in household *i*, we compared 68 candidate models testing different measures of this effect, focusing on measures of effect duration. The best-fitting models in both S-2013 and L-2014 were those that used a weighted value for the number of days since the most recent spray event, or ${a}_{it}=max\left(f\left(\Delta {t}_{c}\right)\right)$ (Fig. 2A).

The temporal weights that resulted in the best-fitting models common to both experiments were Gaussian functions where $\sigma =15-25$ and exponential functions where $k=0.04-0.06$. These decay curves follow a similar trend where the spray effect gradually wanes to a point where sprays that occurred between 10–35 days ago were assigned half the value as a spray that on the same day (Fig. 2B).

The model with the lowest average WAIC rank across the two experiments (${m}_{best}$) applied weights to the time since the most recent spray event according to a Gaussian function with $\sigma =20$, that is

11 $$\begin{array}{c}{f}_{Gaussian,\sigma =20}\left(\Delta {t}_{c}\right)=max\left({e}^{-\frac{\Delta {t}_{c}^{2}}{2\times {20}^{2}}}\right)\end{array}$$

The estimated model coefficients were not equivalent between the two experiments: in S-2013, the estimated incidence rate ratio of the spray event weighted by ${f}_{Gaussian,\sigma =20}\left(\Delta {t}_{c}\right)$ was 0.17 (95% CI [0.10,0.30]), while in L-2014 it was 0.37 (95% CI [0.30,0.45]). We selected the model fit to the L-2014 experiment as the consensus model, as the experimental design in L-2014 allowed for an improved measurement of the effect of sprays occurring < 7 days before adult mosquito collections. The resulting model for L-2014 was:

12 $$\begin{array}{c}{m}_{best}:log\left({\mu }_{it}\right)=-0.906-0.997\times max\left({e}^{-\frac{\Delta {t}_{c}^{2}}{2\times {20}^{2}}}\right)+{\gamma }_{i}+{\delta }_{t}+{\eta }_{i}\end{array}$$

**Fig. 3:**
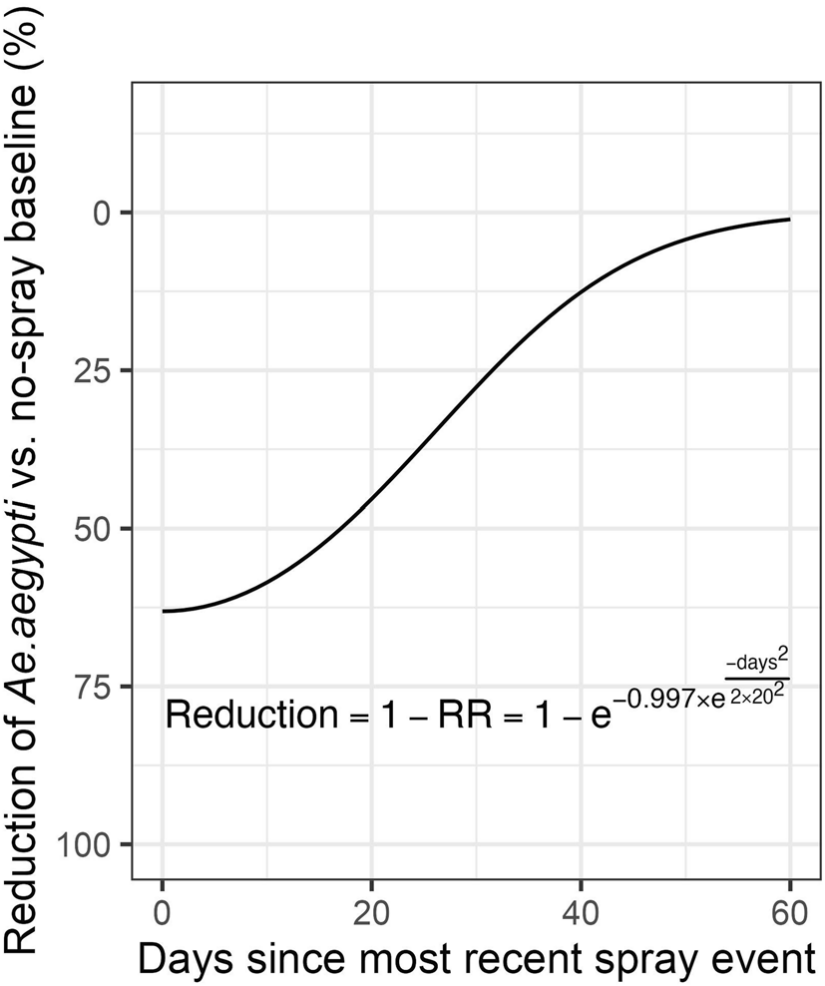
Estimated reduction in the number of *Ae. aegypti* per household as a function of the number of days since the most recent spray event$.$ The equation shown expresses the reduction as a proportion, and rate ratio (RR) is the ratio of rates comparing spray scenarios vs. a no-spray baseline

A rate ratio of 0.37 (0.30, 0.45) suggests that there was a 63% reduction in adult *Ae. aegypti* females in households sprayed on the same day compared to households sprayed > 50 days ago (approximately the difference between values of 1 and 0 in the weight of the time since the most recent spray event according to ${f}_{Gaussian,\sigma =20}\left(\Delta {t}_{c}\right)$) (Fig. 3).

#### Cumulative effects of sequential sprays

There was no improvement of model fit by using the cumulative value of the temporal weights of all previous spray events (Fig. 2A). In addition, when using the same decay function to assign temporally lagged weights to spray events ($f\left(\Delta {t}_{c}\right)$), the estimated rate ratio for spray effects that represented the cumulative weight of all previous spray events ($\sum_{c=1}^{6}\left(f\left(\Delta {t}_{c}\right)\right))$ was consistently higher than the rate ratio of spray effects that assigned a weight only to the most recent spray event (${\text{max}}(f\left(\Delta {t}_{c}\right)$, possibly because of a dilution of weight of the most recent spray effect. Additional file 1: Fig. S4). This indicated that the cumulative effect of sequential sprays in a household had less impact on the reduction in adult *Ae. aegypti* than the effect of the most recent spray. These results suggested that the spray effect was not cumulative over time, and the measurable effects of the spray intervention on the number of female *Ae. aegypti* depended on the most recent spray event.

#### Comparison of within-household spray effect measurements using weighted vs. non-weighted variables

Models using less complex measures of treatment status (i.e. binary or discrete) generally yielded poorer fits to the data compared to models that assigned a weighted value to the time since the most recent spray event using a decay function. Only models that included yes/no variables that indicated if household *i* had been sprayed in the previous 1–4, 1–5 and 1–6 weeks prior to *t* were among the 11 best fitting models along with models that used decay functions. (Additional file 1: Fig. S5).

### Effects of sprays in neighboring households

Models that included a measure for the effect of spraying the households surrounding household *i* (*b*) did not have a significantly improved fit compared to models that only included the measure of the spray effect on household *i* selected in ${m}_{best}$ (${f}_{Gaussian,\sigma =20}\left(\Delta {t}_{c}\right))$. This was evidenced by a lack of a significant difference between the WAIC value of these models and the WAIC value of ${m}_{best}$ (Additional file 1: Fig. S6). This suggests that there was no additional reduction in *Ae. aegypti* that resulted from sprays that occurred in surrounding households.

### Prior sensitivity analysis

A sensitivity analysis on prior specifications confirmed that the selected priors produced consistent results (WAIC and rate ratios) with models that used ten alternative prior specifications for the fixed and random effects (Additional file 1: Fig. S1). This was true for models that included only the effect of spray within a household (*a*) and for models that included an additional effect for sprays in the surrounding houses (*b*). Three of the tested alternative prior specifications resulted in different WAIC and/or rate ratios than the majority, but these were not used to specify the candidate models.

### Estimated recovery profile for *Ae. aegypti* abundance following sprays

The spray effect selected in ${m}_{best}$, ${f}_{Gaussian,\sigma =20}\left(\Delta {t}_{c}\right)$, was used to estimate the reduction in the number of *Ae. aegypti* per household based on the number of days since the last spray event occurred in the household (Fig. 3). The estimated number of *Ae. aegypti* per household, $\mu$, was averaged over the spatial, temporal and household variation (i.e., with each set equal to their mean of zero), thus the rate ratio (RR) comparing spray scenarios with a no-spray baseline was calculated using the remaining terms from ${m}_{best}$:

13 $$\begin{array}{c}Reduction=1-RR= 1- {e}^{-0.997\times {e}^{-\frac{day{s}^{2}}{2\times {20}^{2}}}}\end{array}$$

Estimates given by this model suggest that the spray effect was reduced by 50% approximately 28 days after a spray event, and a near-complete recovery of the *Ae. aegypti* population was reached around 50-60 days post spray.

## Discussion

In this study, we characterized the effect of ULV indoor pyrethroid spraying on the number of *Ae. aegypti* within households in relation to the spray events occurring in the proximity of that household in time and space. Improved understanding of the duration and spatial reach of the effect of a spray intervention on *Ae. aegypti* populations can help to define optimal targets for spatial coverage and frequency of sprays needed during vector control interventions as well as inform modeling efforts that contrast different potential vector control strategies. Our results indicate that the reduction of the *Ae. aegypti* population in a household is due to spray events within that same household, with no additional effect of sprays in households in the neighboring area. The effect of a spray event on the number of *Ae. aegypti* within a household is mainly determined by the time elapsed since the most recent spray event and wanes gradually over the span of 60 days. No added reduction in *Ae. aegypti* numbers was observed from the cumulative effect of multiple past sprays within a household. In summary, the reduction of *Ae. aegypti* in a household is determined mainly by the time since the last spray intervention in that same household.

An important limitation of our study is that we did not account for the age of collected adult *Ae. aegypti*. The previous analysis of these experiments [14] reported a shift to a younger age distribution of adult females (an increased proportion of nulliparous females) in the L-2014 spray zone compared to the buffer zone. Thus, while we did not find an additional explanatory effect of the spray events in surrounding households on the number of *Ae. aegypti* in a given household, we cannot establish that there are not area-wide effects in the population dynamics of the *Ae. aegypti* population in an area where sprays occur regularly.

Other limitations of our study include not being able to account for the MoH emergency sprays that occurred approximately 2 months before the experimental sprays in L-2014 because of the lack of detailed information regarding their location and time. Previous analyses indicate that these sprays had a similar effect on the whole study area, creating a common baseline of *Ae. aegypti* density; indeed, the number of *Ae. aegypti* had started to recover by the time the experimental sprays were implemented [14]. In addition, differences in the results between the two experimental periods could be due to differences in the study design and *Ae. aegypti* susceptibility to cypermetherin, which was greater in S-2013 than in L-2014 [14]. We reported results that found the greatest consensus between the two studies and of those selected the model fit to the L-2014 experiment as our final model. Given that the experimental design in L-2014 was better suited to evaluate the effect of very recent sprays on the number of *Ae. aegypti* in a household, and that the local *Ae. aegypti* population presented resistance to pyrethroids in late 2014 [41], we consider this model to be the more conservative choice that is better suited to address the aims of this work.

Our observations of a waning of the spray effect to a 50% efficacy after 28 days, as well as a 63% reduction in the adult *Ae. aegypti* on the day a household is sprayed compared to if a spray had occurred 60 days ago, are in agreement with the previous analysis of this study, which found that adult mosquito populations partially recovered within 2 weeks of the last spray event, while providing a more nuanced characterization of the population rebound that can be included in future modeling efforts [14]. These results also align with previous studies measuring the effect over time of ULV indoor sprays, where mosquito population recoveries ranged from 50% after seven days after a permethrin mix application [18] to 100% between 14 days to 6 months after an application of organophosphates [42] or 3 weeks with lambda-cyhalothrin [43]. While differences in location, study design, building materials, insecticide product, application method and insecticide resistance may account for the variability of the spray effect duration in these reports, our results fall within a plausible time frame. When comparing models that assigned a weighted value to the spray effect based on a decay function with more easily interpretable binary and discrete measures, we found that only models that included yes/no variables indicating if a household had been sprayed in the previous 1–4, 1–5 and 1–6 weeks were comparable in model fit. While these measures may be easier to interpret, they seem of less value to vector control agencies, as the application of spray interventions over consecutive weeks may not be common practice in many areas and therefore pose little advantage to the use of a weighted measure of the most recent spray event using a Gaussian decay function as described in the results.

The relatively shallow slope of the spray effect decay we observed in this study could be a result of cypermethrin degradation rates combined with mosquito population dynamics. The insecticide applied in this study, cypermethrin, is a pyrethroid that degrades mainly through photolysis and hydrolysis (DT50 = 2.6–3.6 days) [44]. While pyrethroids are generally thought to degrade quickly after application with minimal residuals, pyrethroid degradation rates indoors are much lower than outdoors, and there are several studies that point to cypermethrin remaining in the air and dust inside households for months after spray applications [45–47]. Households in Iquitos often are built as dark narrow corridors with minimal windows, which could explain a decreased degradation rate due to photolysis [14]. In addition, cypermethrin is highly toxic to susceptible *Ae. aegypti* at low doses (LD50 ≤ 0.001 ppm) [48]. Residual cypermethrin is unlikely to affect aquatic immature mosquito forms because of its hydrophobic nature, thus explaining the recovery of adults over time emerging from active larval habitats, demonstrated by the higher proportion of nulliparous females in the spray sector than in the buffer sector described in the original study [14]. The life cycle of an *Ae. aegypti* mosquito from egg to adult can take between 7 to 10 days depending on temperature and mosquito strain [49]. Residual cypermethrin killing or expelling some of newly emerged and imported adults from unsprayed areas, as well as reduced egg-laying due to the decreased number of adults, could further explain the delayed recovery of the adult mosquito population [22, 50].

Models that accounted for the entire history of past sprays in households yielded poorer model fits and weaker estimates of effects compared to models that accounted only for the time since the most recent spray. This should not be taken as evidence that re-treatment of individual households is not necessary. The rebound in *Ae. aegypti* numbers shortly after a spray event observed in our study as well as in the previous study [14] suggests the need for re-treatment of households at a frequency guided by local transmission dynamics to restore the suppression of *Ae. aegypti*. Spray frequency should prioritize reducing the potential for infected *Ae. aegypti* females, which will depend on the expected duration of the extrinsic incubation period (EIP), that is, the time needed for a vector that has ingested an infected blood meal to become infectious to the next host. EIP in turn will depend on virus strain, temperature and other factors [51–53]. For example, in the case of DENV, even if a spray intervention eliminated all infected adult vectors, the human population could still remain infectious for up to 14 days and could infect newly emerged mosquitoes [54]. To control dengue transmission, sprays should occur at intervals shorter than the EIP to eliminate newly emerged mosquitoes that may have bitten infected hosts before they become infectious to others. A period of 7 days can be used as a rule of thumb and a unit that is manageable by vector control agencies. Thus, spraying insecticide every week for at least 3 weeks (to fully cover the infectious period of the host) would be sufficient to prevent DENV transmission, and our results suggest that the efficacy of the previous spray would not have waned considerably at that point [13]. Indeed, in Iquitos, health authorities have successfully reduced DENV transmission during outbreaks by performing three cycles of indoor ULV space spraying with an adulticide over the course of several weeks to months [15].

Lastly, our results indicate that the effect of an indoor spray event is limited to the household where it occurs, and there was no added reduction of *Ae. aegypti* that was attributable to sprays in neighboring households. Adult *Ae. aegypti* likely remain in the vicinity or inside the household where they emerge, clustering within a distance of 10 m and with a mean distance traveled of 106 m [36]. Thus, spraying in the area surrounding a household might not have a large effect on the number of *Ae. aegypti* in that household. This confirms previous findings that found no effect of spraying outside or surrounding a household [18, 55]. However, as stated above, there may be area-wide effects on *Ae. aegypti* population dynamics that our models were not designed to detect.

Together, our results highlight the importance of reaching every household where there is high risk of transmission during an outbreak period, as households without a recent spray intervention cannot rely on nearby interventions or even multiple past interventions to decrease the mosquito population in the present moment. Initial spraying efforts always achieve partial coverage due to inaccessibility of some households (e.g. due to absence or residents or unwillingness to allow treatment). Return visits to missed households can increase coverage but with diminishing returns and higher cost per household with each successive round of attempts. Therefore, improved targeting of vector control programs to areas at higher risk of dengue transmission is needed. Dengue transmission is heterogeneous in space and time, and local evaluations of high-risk areas that include demographic, environmental and social conditions should drive targeted vector control efforts [56, 57]. Other targeting strategies, such as combining indoor residual spraying with contact tracing, have been effective in the past and could be successful in some contexts [56]. Mathematical models can also aid in choosing the best vector control strategy to reduce transmission for each local context, without the need for expensive and logistically challenging field trials [14, 22, 24]. Our results provide detailed parametrizations of the effect of indoor ULV sprays in space and time that can inform future mechanistic modeling efforts.

Success of an insecticide application depends on multiple factors such as the insecticide used, the resistance to that insecticide in the vector population, the local context of household construction, weather variables and method of implementation (including droplet size and spray implement). While our results are specific to the context of this study, they provide nuance to the spatial and temporal effect of a ULV indoor spray intervention that can help inform future planning efforts and modeling studies. Vector control measures and modeling efforts should nonetheless consider the place-specific human, environmental, virus and vector dynamics.

## Conclusions

Taken together, our results suggest that the time since the most recent ULV spray intervention determines the number of *Ae. aegypti* within households, with no cumulative benefit provided by multiple sprays in that household or sprays in the surrounding households. *Aedes aegypti* numbers recover gradually over the course of several weeks. Thus, we recommend that vector control programs focus on maximizing the percentage of households sprayed in high-risk areas and determine the frequency of sprays based on context-specific viral transmission metrics such as the extrinsic incubation period of the local mosquito population under current conditions.

### Supplementary Information

Additional file 1.

## Data Availability

Deidentified data sets are available upon request to the corresponding author according to the program funder data access and sharing policy. Data are located in a secure database located at the University of California, Davis. Code for the data curation, analysis and visualization can be found at 10.5281/zenodo.11244560.

## References

[1] Harrington LC, Edman JD, Scott TW (2001). Why do female *Aedes aegypti* (Diptera: Culicidae) feed preferentially and frequently on human blood?. J Med Entomol.

[2] Scott TW, Takken W (2012). Feeding strategies of anthropophilic mosquitoes result in increased risk of pathogen transmission. Trends Parasitol.

[3] Edman JD, Kittayapong P, Day JF (1997). A fitness advantage for *Aedes aegypti* and the viruses it transmits when females feed only on human blood. Am J Trop Med Hyg.

[4] Ponlawat A, Harrington LC (2005). Blood feeding patterns of *Aedes aegypti* and *Aedes albopictus* in Thailand. J Med Entomol.

[5] Kraemer MUG, Sinka ME, Duda KA (2015). The global distribution of the arbovirus vectors *Aedes aegypti* and *Ae. albopictus*. eLife.

[6] Brady OJ, Gething PW, Bhatt S (2012). Refining the global spatial limits of dengue virus transmission by evidence-based consensus. PLoS Negl Trop Dis.

[7] Bhatt S, Gething PW, Brady OJ (2013). The global distribution and burden of dengue. Nature.

[8] Horstick O, Runge-Ranzinger S, Nathan MB, Kroeger A (2010). Dengue vector-control services: how do they work? A systematic literature review and country case studies. Trans R Soc Trop Med Hyg.

[9] Chadee DD, Tikasingh ES, Ganesh R (1992). Seasonality, biting cycle and parity of the yellow fever vector mosquito *Haemagogus janthinomys* in Trinidad. Med Vet Entomol.

[10] Esu E, Lenhart A, Smith L, Horstick O. Effectiveness of peridomestic space spraying with insecticide on dengue transmission; systematic review. Trop Med Int Health. 2010;15(5). 10.1111/j.1365-3156.2010.02489.x. 10.1111/j.1365-3156.2010.02489.x 20214764

[11] Morrison AC, Zielinski-Gutierrez E, Scott TW, Rosenberg R (2008). Defining challenges and proposing solutions for control of the virus vector *Aedes aegypti*. PLoS Med.

[12] Reiter P, Gubler DJ, Ooi EE, Vasudevan S, Farrar J (2014). Surveillance and control of urban dengue vectors. Dengue and dengue hemorrhagic fever.

[13] Bonds JAS (2012). Ultra-low-volume space sprays in mosquito control: a critical review. Med Vet Entomol.

[14] Gunning CE, Okamoto KW, Astete H (2018). Efficacy of *Aedes aegypti* control by indoor ultra low volume (ULV) insecticide spraying in Iquitos. Peru PLoS Negl Trop Dis.

[15] Stoddard ST, Wearing HJ, Reiner RC (2014). Long-term and seasonal dynamics of dengue in Iquitos, Peru. PLoS Negl Trop Dis.

[16] Achee NL, Grieco JP. Current status of spatial repellents in the global vector control community. In: Corona C, Debboun M, Coats J, editors. Advances in Arthropod Repellents. Elsevier; 2022; p. 267–278. 10.1016/B978-0-323-85411-5.00009-1.

[17] Reiner RC, Achee N, Barrera R (2016). Quantifying the Epidemiological Impact of Vector Control on Dengue. PLoS Negl Trop Dis.

[18] Koenraadt CJM, Aldstadt J, Kijchalao U (2007). Spatial and temporal patterns in the recovery of *Aedes aegypti* (Diptera: Culicidae) populations after insecticide treatment. J Med Entomol.

[19] Castro M, Quintana N, Quiñones PML (2007). Evaluación de dos Piretroides en el control del vector del dengue en Putumayo, Colombia. Rev Salud Pública.

[20] Bowman LR, Runge-Ranzinger S, McCall PJ (2014). Assessing the relationship between vector indices and dengue transmission: a systematic review of the evidence. PLoS Negl Trop Dis.

[21] Runge-Ranzinger S, Horstick O, Marx M, Kroeger A (2008). What does dengue disease surveillance contribute to predicting and detecting outbreaks and describing trends?. Trop Med Int Health.

[22] Cavany SM, España G, Lloyd AL (2020). Optimizing the deployment of ultra-low volume and targeted indoor residual spraying for dengue outbreak response. PLOS Comput Biol.

[23] Hladish TJ, Pearson CAB, Patricia Rojas D (2018). Forecasting the effectiveness of indoor residual spraying for reducing dengue burden. PLoS Negl Trop Dis.

[24] Gunning CE, Morrison AC, Okamoto KW (2022). A critical assessment of the detailed *Aedes aegypti* simulation model Skeeter Buster 2 using field experiments of indoor insecticidal control in Iquitos. Peru PLoS Negl Trop Dis.

[25] Morrison AC, Astete H, Chapilliquen F (2004). Evaluation of a sampling methodology for rapid assessment of *Aedes aegypti* infestation levels in Iquitos, Peru. J Med Entomol.

[26] Vazquez-Prokopec GM, Galvin WA, Kelly R, Kitron U (2009). A new, cost-effective, battery-powered aspirator for adult mosquito collections. J Med Entomol.

[27] Green JA (2021). Too many zeros and/or highly skewed? A tutorial on modelling health behaviour as count data with Poisson and negative binomial regression. Health Psychol Behav Med.

[28] Lindgren F, Rue H Bayesian Spatial Modelling with *R* - INLA. J Stat Softw. 2015; 10.18637/jss.v063.i19.

[29] Lindgren F, Rue H, Lindström J (2011). An explicit link between Gaussian fields and Gaussian Markov random fields: the stochastic partial differential equation approach. J R Stat Soc Ser B Stat Methodol.

[30] Moraga P. Geospatial Health Data: Modeling and Visualization with R-INLA and Shiny. Chapman & Hall/CRC Biostatistics Series. 2019.

[31] Rue H, Martino S, Chopin N. R-INLA—Documentation for Random Walk Model of Order 1 (RW1). 2023. https://inla.r-inla-download.org/r-inla.org/doc/latent/rw1.pdf. Accessed 16 Nov 2023.

[32] Rue H, Martino S, Chopin N (2009). Approximate Bayesian inference for latent Gaussian models by using integrated nested Laplace approximations. J R Stat Soc Ser B Stat Methodol.

[33] Simpson DP, Rue H, Martins TG, et al. Penalising model component complexity: A principled, practical approach to constructing priors. 2015.

[34] Watanabe S (2013). A widely applicable Bayesian information criterion. J Mach Learn Res.

[35] Gelman A, Hwang J, Vehtari A (2014). Understanding predictive information criteria for Bayesian models. Stat Comput.

[36] Moore TC, Brown HE (2022). Estimating *Aedes aegypti* (Diptera: Culicidae) flight distance: meta-data analysis. J Med Entomol.

[37] Marcantonio M, Reyes T, Barker CM. Quantifying *Aedes aegypti* dispersal in space and time: a modeling approach. Ecosphere. 2019;10(12). 10.1002/ecs2.2977.

[38] Getis A, Morrison AC, Gray K, Scott TW (2003). Characteristics of the spatial pattern of the dengue vector, *Aedes aegypti*, in Iquitos, Peru. Am J Trop Med Hyg.

[39] Edman JD, Kittayapong P, Coleman RC (2005). Dispersal of the dengue vector *Aedes aegypti* within and between rural communities. Am J Trop Med Hyg.

[40] Harrington LC, Buonaccorsi JP, Edman JD (2001). Analysis of survival of young and old *Aedes aegypti* (Diptera: Culicidae) from Puerto Rico and Thailand. J Med Entomol.

[41] Palomino-Salcedo M. Estado de susceptibilidad de la poblacion natural de *Aedes aegypti* a los insecticidas en Punchana-Iquitos, Region Loreto (Noviembre 2014). Instituto Nacional de Salud, Perú. 2014.

[42] Pant CP, Mathis HL, Nelson MJ, Phanthumachinda B (1974). A large-scale field trial of ultra-low-volume fenitrothion applied by a portable mist blower for the control of *Aedes aegypti*. Bull World Health Organ.

[43] Perich MJ, Rocha NO, Castro AL (2003). Evaluation of the efficacy of lambda-cyhalothrin applied by three spray application methods for emergency control of *Aedes aegypti* in Costa Rica. J Am Mosq Control Assoc.

[44] Working Group E of the Common Implementation Strategy for the Water Framework Directive—European Commission. Cypermethrin Environmental Quality Standard Dossier. 2011. https://circabc.europa.eu/sd/a/86977d7a-36b1-4c92-adf4-d403a75ce623/Cypermethrin%20EQS%20dossier%202011.pdf. Accessed 23 Jun 2023.

[45] Tang W, Wang D, Wang J (2018). Pyrethroid pesticide residues in the global environment: An overview. Chemosphere.

[46] Wright CG, Leidy RB, Dupree HE (1993). Cypermethrin in the ambient air and on surfaces of rooms treated for cockroaches. Bull Environ Contam Toxicol.

[47] Leng G, Berger-Preiß E, Levsen K (2005). Pyrethroids used indoor - ambient monitoring of pyrethroids following a pest control operation. Int J Hyg Environ Health.

[48] Hribar LJ, Murray HL (2019). Toxicity of Tau-Fluvalinate, Lambda-Cyhalothrin, and Alpha-Cypermethrin to *Aedes aegypti*, 2019. Arthropod Manag Tests..

[49] Arévalo-Cortés A, Granada Y, Torres D, Triana-Chavez O (2022). Differential hatching, development, oviposition, and longevity patterns among Colombian *Aedes aegypti* populations. Insects.

[50] Sathantriphop S, Paeporn P, Ya-umphan P (2020). Behavioral action of deltamethrin and cypermethrin in pyrethroid-resistant *Aedes aegypti* (Diptera: Culicidae): implications for control strategies in Thailand. J Med Entomol.

[51] Lambrechts L, Paaijmans KP, Fansiri T (2011). Impact of daily temperature fluctuations on dengue virus transmission by *Aedes aegypti*. Proc Natl Acad Sci.

[52] Mordecai EA, Cohen JM, Evans MV (2017). Detecting the impact of temperature on transmission of Zika, dengue, and chikungunya using mechanistic models. PLoS Negl Trop Dis.

[53] Chan M, Johansson MA (2012). The incubation periods of dengue viruses. PLoS ONE.

[54] Reiter PNMB. Guidelines for assessing the efficacy of insecticidal space sprays for control of the dengue vector *Aedes aegypti*. World Health Organ. 2001.

[55] Sudsom N, Techato K, Thammapalo S (2020). Indoor spray and windows screens effects on dengue vector density after space spraying in a field trial. Asian Pac J Trop Med.

[56] Vanlerberghe V, Gómez-Dantés H, Vazquez-Prokopec G, Alexander N, Manrique-Saide P, Coelho G (2017). Changing paradigms in *Aedes* control: considering the spatial heterogeneity of dengue transmission. Rev Panam Salud Publica.

[57] Liebman KA, Stoddard ST, Morrison AC (2012). Spatial dimensions of dengue virus transmission across interepidemic and epidemic periods in Iquitos, Peru (1999–2003). PLoS Negl Trop Dis.

