# Supplementary material for: Spatial and temporal analysis on the impact of ultra-low volume indoor insecticide spraying on Aedes aegypti household density
Source: Parasit Vectors. 2024 Jun 11;17:254. doi: 10.1186/s13071-024-06308-3 (PMC11165869; doi:10.1186/s13071-024-06308-3)
Supplement: Supplementary file 1 — Additional file 1. [file 13071_2024_6308_MOESM1_ESM.pdf]

1 Prior sensitivity analysis

All candidate models in this study were run using default uninformative prior distributions recommended in the R-INLA documentation, with the exception of the prior for the SPDE Matérn function, where a value of 10 was assigned to the range and of 1 to the standard deviation of the spatial field using the PC prior function; this combination of priors is hereafter named "original". The default priors, as described in the main text, are a zero- mean Gaussian prior distributions $\beta \sim Normal(0, 0.001^{-1})$ for all fixed effects (*a* and *b*) and log gamma prior distributions for the iid and RW1 random effects ($\eta_i \sim loggamma(1, 0.00005)$ and $\delta_t \sim loggamma(1, 0.00005)$). To ensure that other prior choices would not affect the results, we performed a prior sensitivity analysis on both m_{best}, the best fitting model including an effect for spray within the household ($a_{f_{gaussian,\sigma=20}(\Delta t_c)}$), and the best fitting model including *a* and an effect for spray in neighboring households ($a_{f_{gaussian,\sigma=20}(\Delta t_c)}$ + $b_{f_{inverse}(d_{ij})}$). We then assessed the difference in WAIC and rate ratios of the fixed effects when using alternative priors for the fixed and random effects as described in Table 1 (Additional file 1: Table S1). Alternative priors were assigned based on reasonable values for each parameter, as well as values that seemed extreme to asses if these changed the model results. Values for the penalized complexity (PC) priors for the precision (τ) of the random walk of order 1 (RW1) and the independent, identically distributed (iid) hyperparameters were selected by simulating the prior distributions using the inlatools R package to allow for a visual assessment. These PC priors are defined by the parameters σ_0 and α such that $P(\sigma > \sigma_0) = \alpha$. For the range and standard deviation of the spatial field and the fixed effect *a* in each model, the prior values were based on previous knowledge of the flight range of *Ae. aegypti* and the range of the weighted variable *a*.

Table 1: Prior sensitivity analysis

Effect	Varying parameters	Label
original	$range = c(10, 0.01); \sigma = c(1, 0.01)$	original
	$range = c(0.1, 0.01); \sigma = c(1, 0.01)$	spde2.1
γ_i Spatial autocorrelation random effect	$range = c(100, 0.01); \sigma = c(1, 0.01)$	spde2.2
	$range = c(10, 0.01); \sigma = c(0.5, 0.01)$	spde2.3
	$range = c(10, 0.01); \sigma = c(3, 0.01)$	spde2.4
	$\sigma_0 = 1; \alpha = 0.01$	rw1.1
δ_t RW1 random effect	$\sigma_0 = 0.25; \alpha = 0.05$	rw1.2
	$\sigma_0 = 0.5; \alpha = 0.05$	rw1.3
	$\sigma_0 = 0.1; \alpha = 0.05$	iid.1
η_i iid random effect	$\sigma_0 = 1; \alpha = 0.05$	iid.2
	$\sigma_0 = 3; \alpha = 0.05$	iid.3
	mean=0, precision= 10	a.g20.1
β_a Fixed effect of spraying house i at time t	mean=0, precision= 1	a.g20.2
	mean=0, precision= 0.5	a.g20.3

Additional file 1: Figure S1

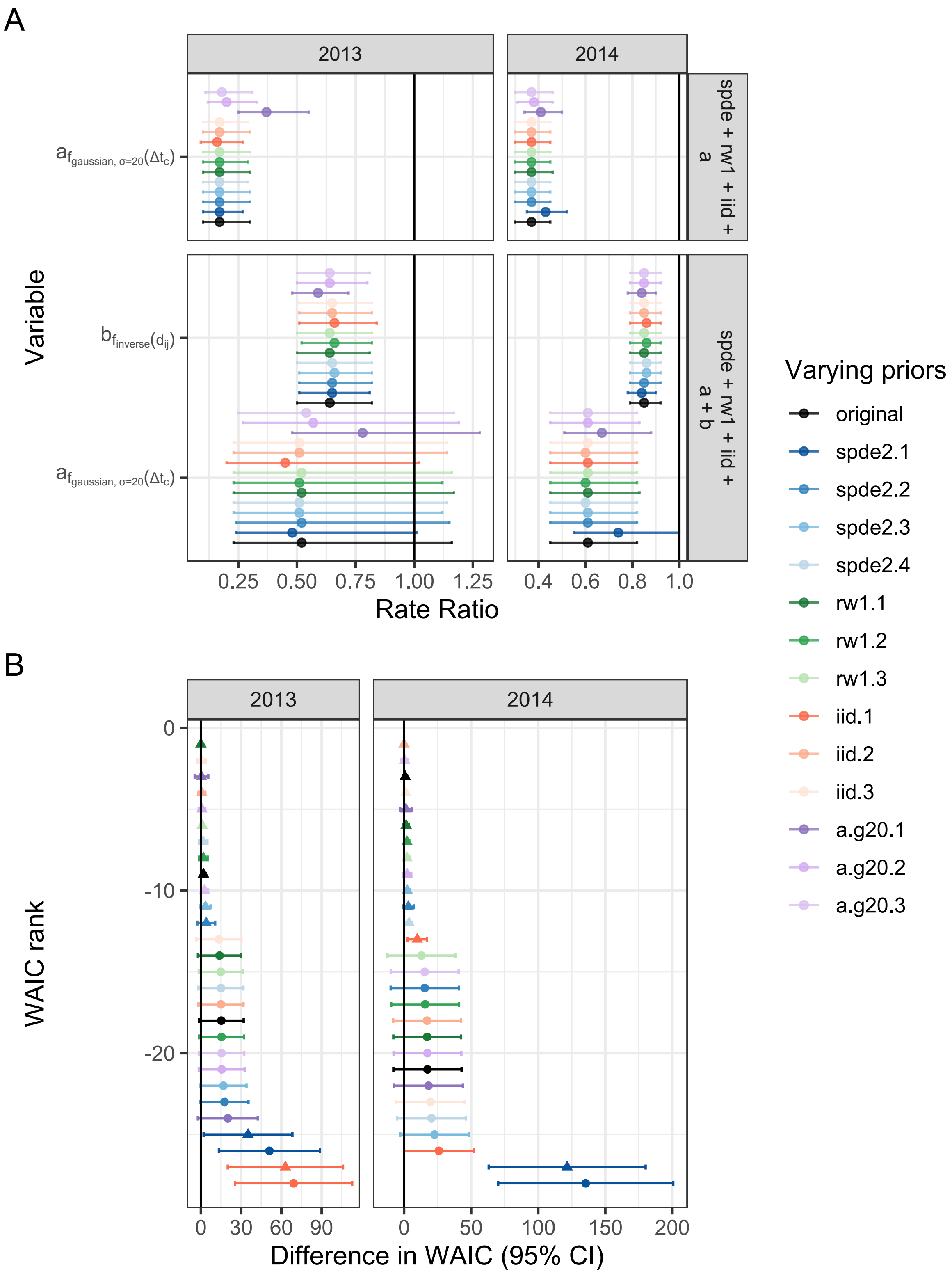

Figure 1: Comparison of model results using alternative priors for fixed and random effects, both in models that only included the effect of spray within a household (a) as well as in models that included an additional effect for sprays in the surrounding houses ($a+b$). A) Prior selection effect on rate ratio. B) Prior selection effect on WAIC.

2 Within-household spray effects (*a*)

Table 2: Discrete and continuous time variables measuring the spray effect on house i

Spray effect	Description
Zone	House in spray zone/house in buffer zone
Sprayed	Was house i sprayed previously? yes/no
Number of sprays	How many times was house i sprayed previously? 1,2...6
Days since the most recent spray	$max(\Delta t_c)$
Weeks since most recent spray	$\frac{max(\Delta t_c)}{7}$
Sprayed 1week prior	Was house i sprayed in the indicated previous week?
Sprayed 2weeks prior	
Sprayed 3weeks prior	
Sprayed 4weeks prior	
Sprayed 5weeks prior	
Sprayed 6weeks prior	
Sprayed 1week prior+ sprayed 2weeks prior	Was house i sprayed in any of the indicated previous weeks?
Sprayed 1week prior+ sprayed 2weeks prior + sprayed 3weeks prior	
Sprayed 1week prior+ sprayed 2weeks prior + sprayed 3weeks prior +sprayed 4weeks prior	
Sprayed 1week prior+ sprayed 2weeks prior + sprayed 3weeks prior +sprayed 4weeks prior + sprayed 5weeks prior	
Sprayed 1week prior+ sprayed 2weeks prior + sprayed 3weeks prior +sprayed 4weeks prior + sprayed 5weeks prior+ sprayed 6weeks prior	

Table 3: A. Weighted time variables measuring the spray effect on house i

Spray effect	Description	Varying parameters
Inverse weight of the most recent spray	$max(\frac{1}{\Delta t_c})$	
Cumulative inverse weight of all previous sprays	$\sum_{c=1}^{6} \frac{1}{\Delta t_c}$	
Gaussian weight of the most recent spray	$max(e^{-\frac{\Delta t_c^2}{2\times\sigma^2}})$	$\sigma = 1, 3, 5, 7, 10, 15, 20, 25, 30, 35, 40, 50, 60, 80$
Cumulative Gaussian weight of all previous sprays	$\sum_{c=1}^{6} e^{-\frac{\Delta t_c^2}{2\times\sigma^2}}$	
Exponential weight of the most recent spray	$max(e^{-(k\times\Delta t_c)})$	$k = 0.005, 0.01, 0.015, 0.02, 0.03, 0.04, 0.06, 0.1, 0.2, 0.4, 1$
Cumulative exponential weight of all previous sprays	$\sum_{c=1}^{6} e^{-(k\times\Delta t_c)}$	

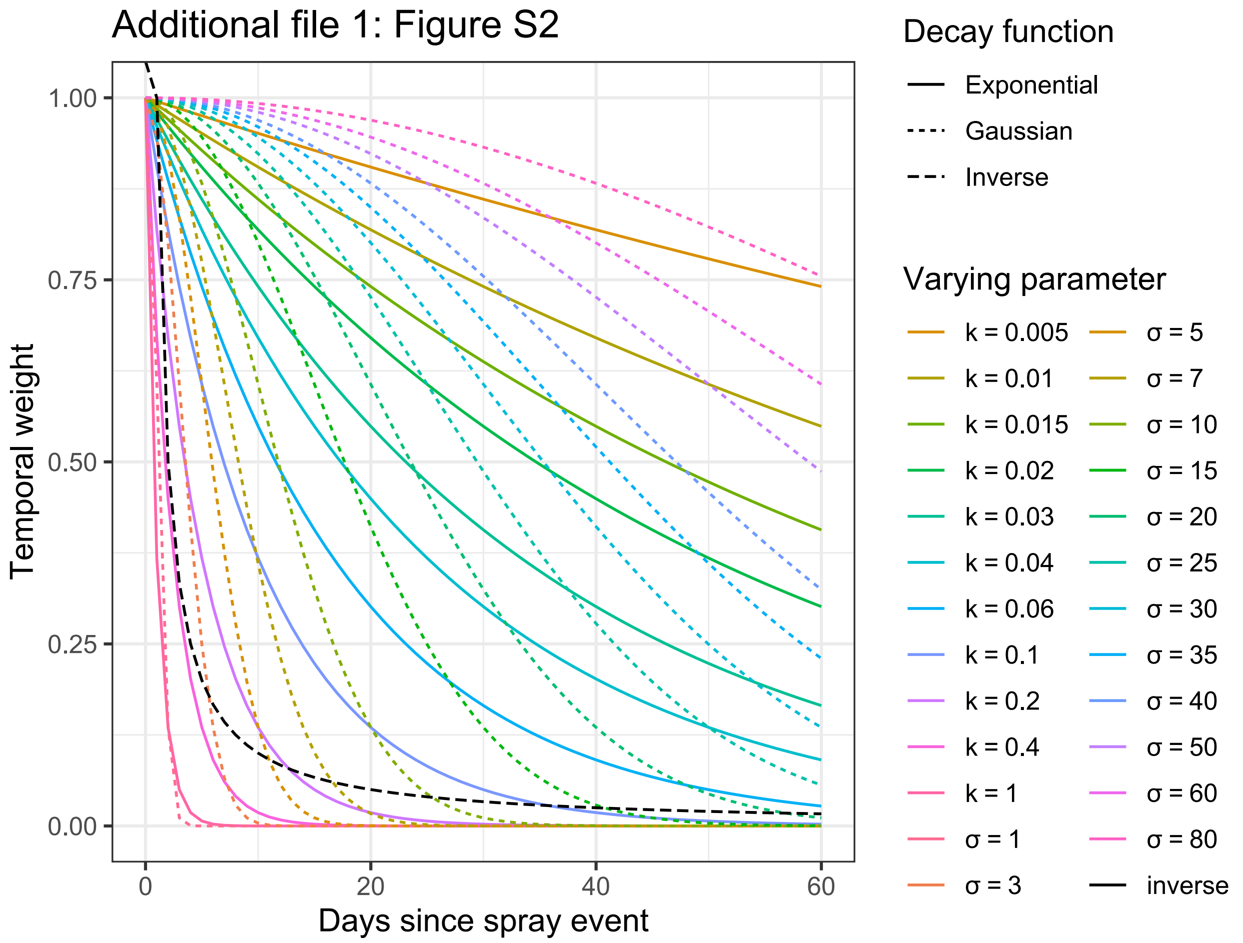

Figure 2: Visual representation of decay functions used to calculate candidate variables that assigned a weight to the days between the date of the adult survey t and the date of a spray event that occurred prior to t (Δt_c).

3 Effects of sprays in neighboring households (*b*)

Table 4: B. Simple spray effects of surrounding houses

Spray effect	Description
% Sprayed 0-100m	Proportion of houses sprayed in the previous week within a ring of a given distance from house i
% Sprayed 0-100m + % sprayed 101-200m	
% Sprayed 0-100m + % sprayed 101-200m + % sprayed 201-300m	
% Sprayed 0-100m + % sprayed 101-200m + % sprayed 201-300m + % sprayed 301-400m	
% Sprayed 0-100m + % sprayed 101-200m + % sprayed 201-300m + % sprayed 301-400m + % sprayed 401-500m	
% Sprayed 0-100m + % sprayed 101-200m + % sprayed 201-300m + % sprayed 301-400m + % sprayed 401-500m + %sprayed >500m	
% sprayed 0-31m	
% Sprayed 0-31m + % sprayed 31-100m	
% Sprayed 0-31m + % sprayed 31-100m + % sprayed 101-300m	
% Sprayed 0-31m + % sprayed 31-100m + % sprayed 101-300m + % sprayed >300m	

Table 5: B. Weighted spray effects of surrounding houses

Spray effect	Description	Varying parameters
Inverse cumulative weight of sprays in the study area	$\sum_j(\frac{1}{d_{ij}}) \times f_{m_{best\ a}}(\Delta t_c)$	
Gaussian cumulative weight of sprays in the study area	$\sum_j(e^{-\frac{d_{ij}^2}{2\times\sigma^2}}) \times f_{m_{best\ a}}(\Delta t_c))$	$\sigma = 5, 25, 50, 75, 100, 125, 150, 200, 250, 300$
Exponential cumulative weight of sprays in the study area	$\sum_j(e^{-(k\times d_{ij})})) \times f_{m_{best\ a}}(\Delta t_c))$	$k = 0.0025, 0.0035, 0.005, 0.0075, 0.01, 0.0125, 0.02, 0.045, 0.2$

Additional file 1: Figure S3

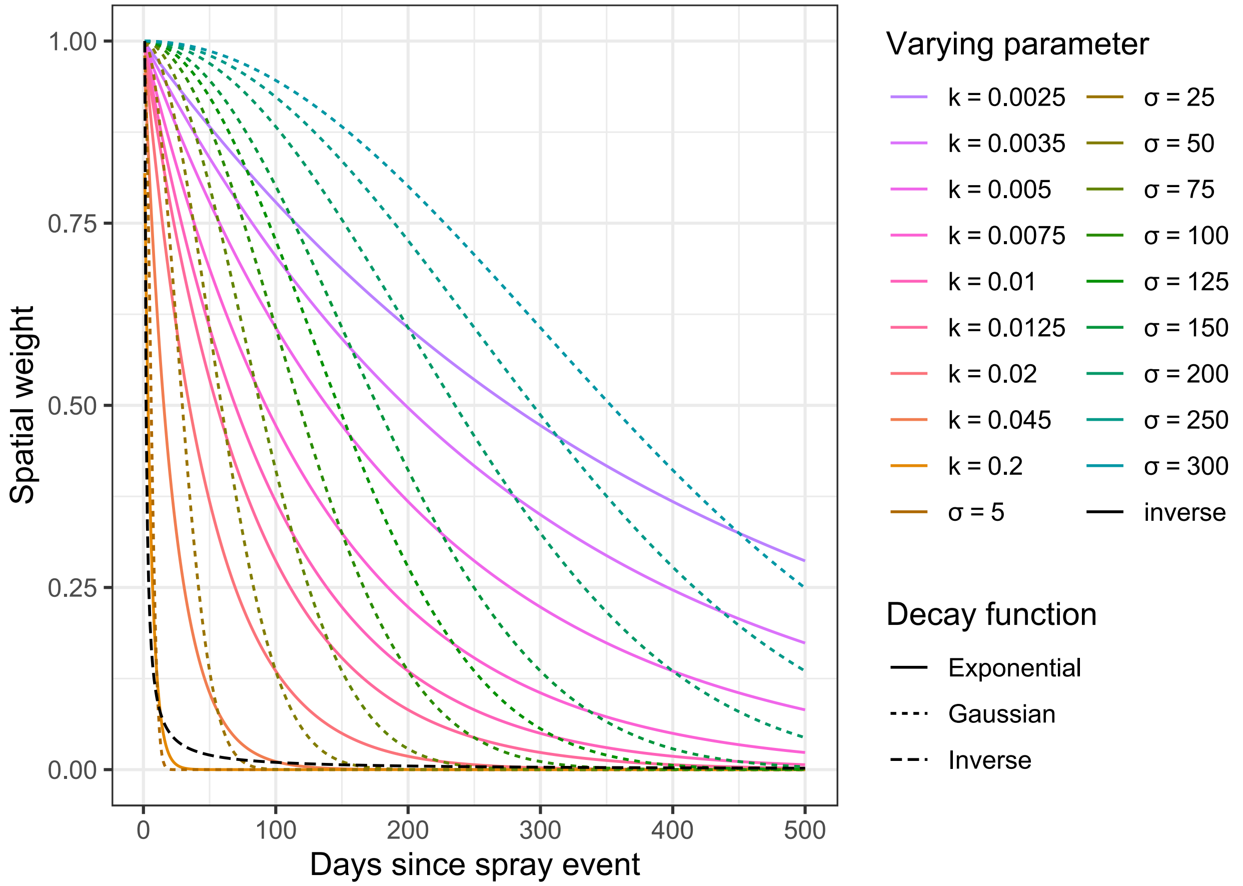

Figure 3: Visual representation of decay functions used to calculate candidate variables that assigned a weight to the distance (in m) between the household i and every surrounding household j (d_{ij})

4 Cumulative effects of sequential sprays

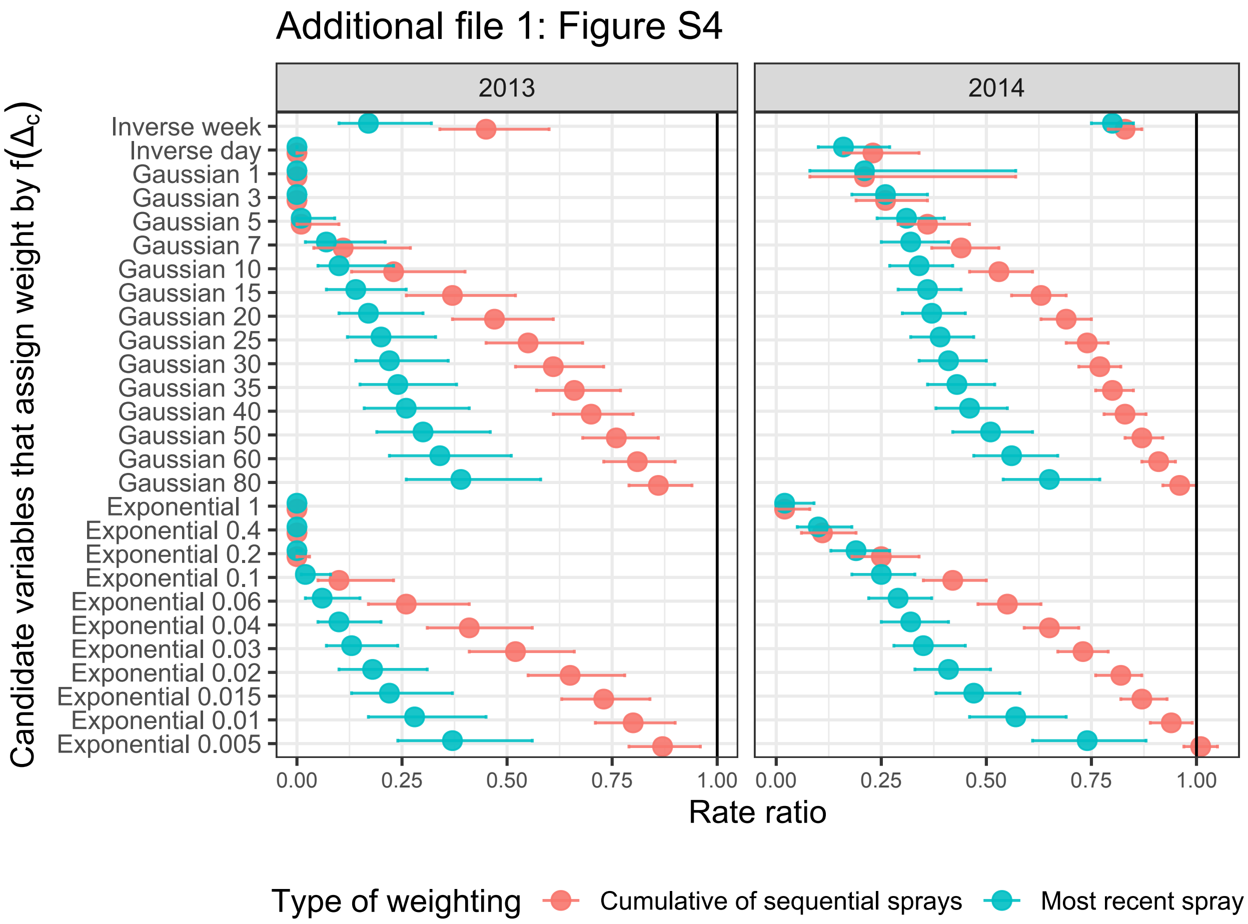

Figure 4: Comparison of the estimated spray effect between models where variable a assigned a weighted value to the most recent spray ($a_{it} = max(f(\Delta t_c))$) (blue) vs. models where variable a was a cumulative value of the weights of all previous spray events ($a_{it} = \sum_6^1(f(\Delta t_c))$) (red).

5 Comparison of within-household spray effect measurements using weighted vs. non-weighted variables

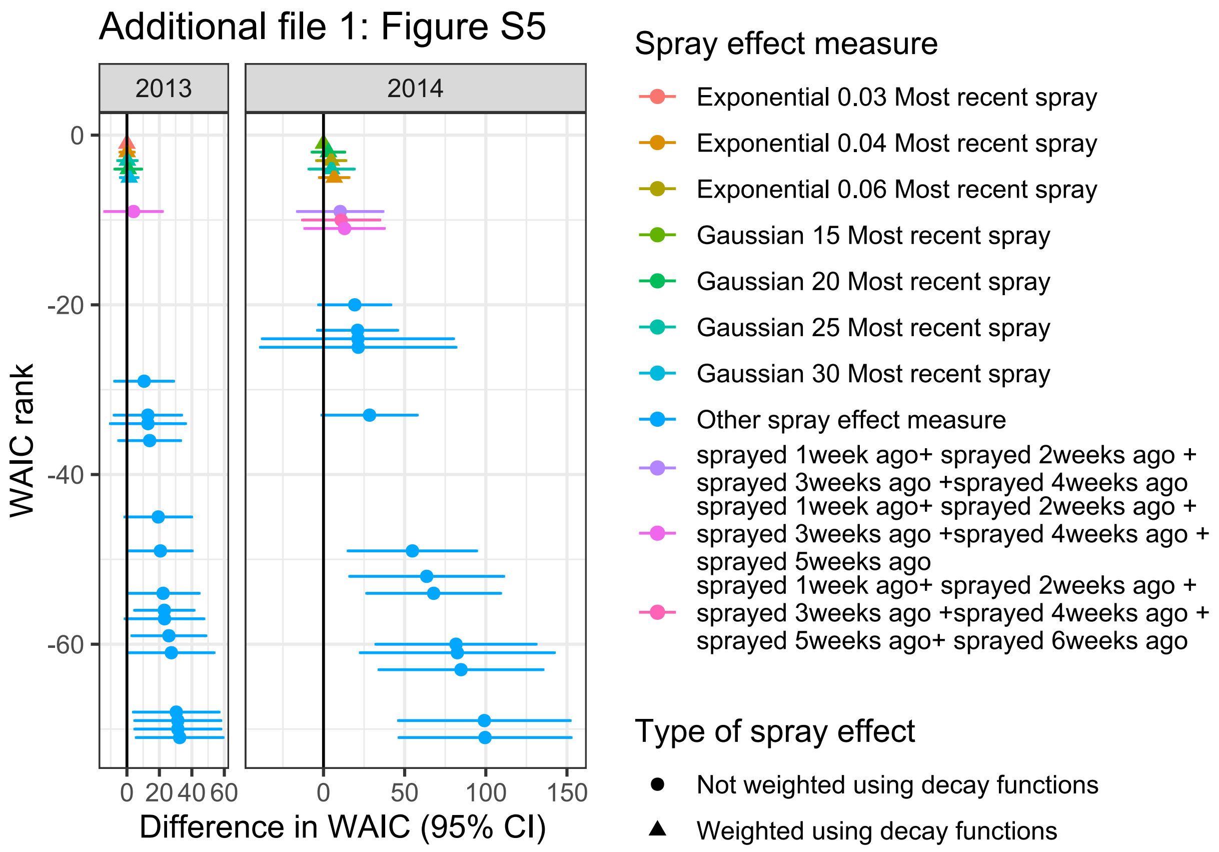

Figure 5: Comparison of WAIC rank between all the models that did not use a decay function to assign a weighted value to Δt_c and the best 5 fitting models. Only the spray effect measures with the top 11 WAIC ranks are named, those labeled 'Other spray effect measure' can be found in Additional file 1: Table S2

6 Effects of sprays in neighboring households

Additional file 1: Figure S6

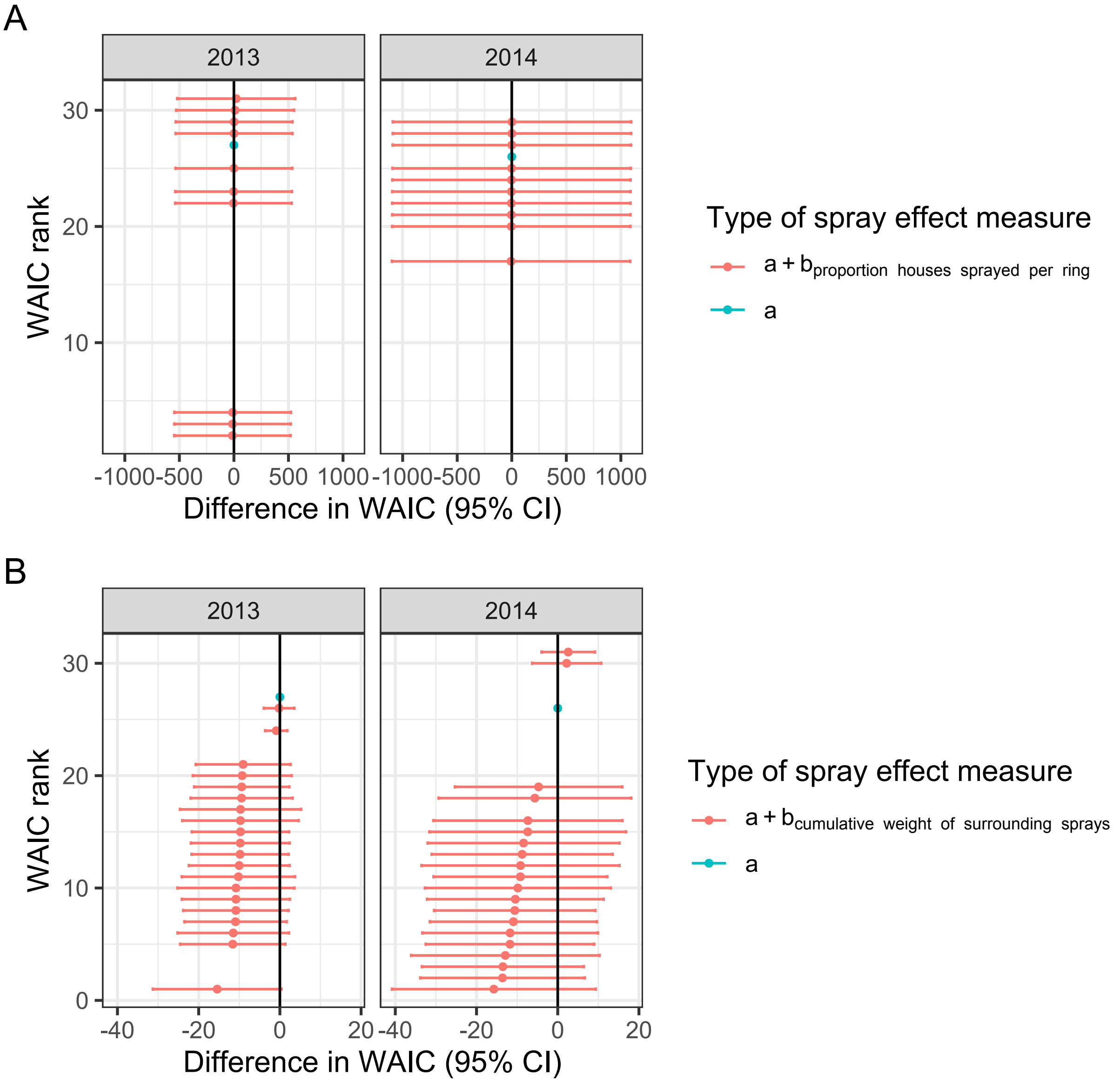

Figure 6: Difference in WAIC between m_{best}, the best fitting model for within-household spray effects, where the spray effect is measured by $a_{f_{gaussian,\sigma=20}(\Delta t_c)}$ (in blue), and models with an additional effect that measures the sprays in the neighboring households (in red). A) The additional effect of sprays in the neighboring households is measured by a proportion of houses sprayed in the previous week within a given distance ring. B) The additional effect of sprays in the neighboring households is measured by a cumulative weight of the distance in time and space since a neighboring house was sprayed for the entire area within 1,000 m of a given household.
